# Safety of von Willebrand factor substitution for neuraxial anesthesia in women with persistent von Willebrand deficiency at delivery

**DOI:** 10.1016/j.rpth.2026.106796

**Published:** 2026-06-12

**Authors:** Yaquine Mechelfekh, Sarah Guérin, Kenza Kali, Pauline Noyel, Aurélie Montmartin, Marie Tuffigo, Maud Colinart-Thomas, Alexandre Butelet, Benoit Guillet, Céline Falaise, Nicolas Drillaud, Miréla Chirila-Hetsch, Alain Ramassamy, Philippe Beurrier, Brigitte Tardy

**Affiliations:** 1 Laboratory of Hematology, Angers University Hospital Center (CHU), Angers, France; 2 Center for Research in Cancerology and Immunology Nantes Angers (CRCI2NA), French National Institute of Health and Medical Research (INSERM), French National Centre for Scientific Research (CNRS), Angers University, Nantes University, Angers, France; 3 Department of Anesthesia and Critical Care, University Hospital of Angers, Angers, France; 4 Clinical Investigation Center (CIC) 1408, French National Institute of Health and Medical Research (INSERM), Saint-Etienne, France; 5 Laboratory of Hematology, Saint-Etienne University Hospital, France; 6 Université Jean Monnet Saint-Étienne, Mines Saint-Etienne, INSERM, SAINBIOSE U1059, Saint-Etienne, France; 7 Center for Rare Bleeding Disorders, Reims University Hospital, Reims, France; 8 Hemophilia Treatment Center, Rennes University Hospital, Rennes, France; 9 Université de Rennes, CHU de Rennes, Inserm, EHESP, Irset (Institut de recherche en santé, environnement et travail)—UMR_S 1085, Rennes, France; 10 Hemostasis Clinical Center, Marseille University Hospital, Assistance Publique-Hôpitaux de Marseille, Marseille, France; 11 Hemophilia Treatment Center, Nantes University Hospital, Nantes, France; 12 Department of Hematology, Valence Hospital, Valence, France; 13 Center for Rare Bleeding Disorders, Poitiers University Hospital, Poitiers, France; 14 Hemophilia Treatment Center, Vascular and Coagulation Clinic, Angers University Hospital, Angers, France; 15 Centre de Traitement de l'Hémophilie, CHU Saint-Etienne, Saint-Etienne, France; 16 Inserm CIC 1408, CHU Saint-Etienne, Saint-Etienne, France; 17 Mines Saint-Etienne, INSERM, SAINBIOSE U1059, Saint-Etienne, France

**Keywords:** von Willebrand diseases, von Willebrand factor, delivery, obstetric, anesthesia, obstetrical, patient safety

## Abstract

**Background:**

Neuraxial anesthesia (NA) constitutes a risk for patients with bleeding disorders, the main hemorrhagic adverse effect being spinal epidural hematoma. No clear recommendation has been issued concerning NA use for delivery in patients with von Willebrand disease (VWD) whose von Willebrand factor (VWF) levels have not been spontaneously corrected by the end of pregnancy.

**Objectives:**

This study describes the experience of 8 French hospital centers with NA use during delivery in these patients.

**Methods:**

Patients included in this study manifested still uncorrected VWF levels at the end of pregnancy and received NA for delivery together with VWF substitution to avoid the risk of hemorrhage associated with this type of anesthesia. Thirty-two patients participated in the study, accounting for 40 pregnancies in total. All VWD types were represented except for type 3. VWF, factor (F)VIII, fibrinogen and platelet levels were recorded before and at the end of pregnancy. The monitoring of VWF levels, the type of VWF ± FVIII substitution, and the doses administered were also noted. We additionally reviewed the literature concerning NA use at delivery in patients with VWD.

**Results:**

No spinal epidural hematoma or ecchymosis related to NA was observed in any of the 32 patients during the 40 deliveries.

**Conclusion:**

In conclusion, our results suggest that patients with VWD manifesting VWF levels not spontaneously corrected by the end of pregnancy could safely benefit from NA with closely monitored VWF substitution. Based on these results and national and international recommendations, we formulated proposals on how to manage these patients.

## Essentials

• Uncorrected VWF at delivery risks bleeding with NA.
• Patients with persistent VWF deficiency received VWF substitution during delivery.
• NA was safely performed in all 40 deliveries without any spinal epidural hematoma or ecchymosis.
• Monitored VWF substitution safely allows NA in VWD deliveries with uncorrected VWF levels.

## 1 Introduction

Von Willebrand disease (VWD) is a hereditary bleeding disorder characterized by deficient or defective von Willebrand factor (VWF) [1]. Symptoms vary from mild bruising to severe bleeding episodes mostly involving the mucosa, particularly the uterine mucosa in women. Bleeding episodes can also occur during childbirth, surgery, or invasive procedures. The clinical expression of VWF deficiency depends on the severity of the defect and its subtype, classified as follows: type 1 (partial quantitative deficiency), type 2 (qualitative defects), and type 3 (near-total deficiency) [2]. Although pregnancy usually causes a physiological elevation of VWF levels [3], VWF activity remains low in some patients even at the end of pregnancy [4].

The use of neuraxial anesthesia (NA) for the delivery of patients with VWD is subject to debate owing to the delicate benefit–risk balance [5]. NA provides effective pain relief and can ease labor but carries a risk of hemorrhage, particularly spinal epidural hematoma (SEH). The pathophysiology of SEH is still not firmly established, but its’ generally accepted cause is a vascular breach in the epidural venous plexus, leading to an accumulation of blood between the vertebra and the dura mater, which may compress the spinal cord [6]. This compression can have dramatic neurologic consequences [7]. The risk of SEH related to NA is low in the general population (1/150,000 for epidural anesthesia and 1/220,000 for spinal anesthesia) [8] but may increase in patients with VWD if VWF levels remain uncorrected at the end of pregnancy.

Patients with VWD in whom spontaneous correction of VWF levels has not occurred by the end of pregnancy receive VWF substitution during labor to avoid the risk of bleeding related to delivery. VWF substitution therapy could at the same time preclude the hemorrhagic risk related to NA. However, this is not widely admitted in general practice, even though the latest international recommendations [9] envisage the use of NA when VWF activity levels are >0.5 IU/mL, whatever the modalities of their normalization (spontaneous or after substitution). These recommendations are nevertheless based on studies not specifically dedicated to VWF deficiency and have a low certainty of evidence.

The aim of this retrospective study was to describe the experience of French hospital centers with NA use in patients with VWF deficiency, considering that well-monitored VWF replacement therapy could avoid both the risk of gynecologic hemorrhage and the hemorrhagic risk related to NA, and to evaluate the safety of this practice.

## 2 Methods

This study comprised a multicenter retrospective review conducted in the 42 French hospital centers possessing a bleeding disorder center (BDC), analyzing in each center patients with VWD with uncorrected VWF deficiency at the end of pregnancy who benefited from NA for delivery after having received VWF concentrates. The hospital pharmacist of each center listed all patients who received VWF concentrates in the maternity ward between 2008 and 2022. The BDC physician checked whether those patients had benefited from NA. A standardized form was used to retrieve clinical data, laboratory results, and delivery data from the medical records.

Our study was conducted in accordance with the ethical standards of the Helsinki Declaration of 1975, as revised in 2008. It was approved by the ethics committee of Saint-Etienne University Hospital Center on January 15, 2023 (IRBN052023/CHUSTE) and by the French data protection agency (2203772). As this was a noninterventional retrospective study, patients received an information letter 1 month before data collection. The study followed the Strengthening the Reporting of Observational Studies in Epidemiology (STROBE) reporting guideline.

### 2.1 Study population

Adult women (> 18 years) with inherited VWD meeting the following 3 inclusion criteria were included:

(1) Presenting VWF deficiency not spontaneously corrected by the end of pregnancy, defined as a VWF:Act level and/or a factor (F)VIII activity (FVIII:C) level of < 0.5 IU/mL at the end of pregnancy.
(2) Having received VWF (±FVIII) concentrate infusions at delivery.
(3) Having benefited from NA.

Patients failing to meet any 1 of the abovementioned 3 inclusion criteria were excluded.

### 2.2 Study variables

Three types of data were retrieved as follows:

- Clinical: age, weight, previous surgery and/or blood transfusion, and/or VWF infusion.
- Laboratory results: VWF:Act, VWF-antigen (VWF:Ag), FVIII:C, fibrinogen levels, and platelet counts at diagnosis and at the end of pregnancy. If available, the occlusion times (OT; by PFA®) were also recorded.
- Delivery data:
  o Delivery mode: vaginal birth or cesarean section, spontaneous or induced labor, occurrence of tears, or need for episiotomy.
  o VWF substitution: VWF concentrate selected, dosage, number of infusions ± FVIII concentrate ± tranexamic acid.
  o Laboratory and obstetrical monitoring:
    - VWF:Act and FVIII:C levels.
    - Blood loss (in milliliters) during delivery, to detect potential postpartum hemorrhage (PPH). Blood losses were objectively quantitated using a graduated under-buttocks collection drape (quantitative blood loss), placed immediately after birth and prior to placental expulsion. This method conforms to international guidelines to prevent the systematic underestimation associated with visual estimation. PPH was strictly defined as a measured blood loss of > 500 mL.
  o Safety of NA: absence of potential adverse effects of anesthesia (ecchymosis at the needle-puncture site, lumbar pain, radicular pain, and motor loss).

### 2.3 Statistical analyses

Descriptive statistics were used for the data set. For continuous variables, median values were used to express the central tendency. Variability was expressed by minimum and maximum values.

Wilcoxon signed-rank test was used to determine whether the median values of VWF:Act, VWF:Ag, and FVIII:C differed between baseline and term (∗*P* < .05; ∗∗*P* < .01; ∗∗∗*P* < .001). Differences were considered significant at *P* < .05. GraphPad Prism 9.5 software was used to analyze the data and generate the figures.

### 2.4 Literature review

A literature review concerning the use of NA at delivery in patients with VWD was performed. PubMed and Google Scholar electronic databases were used to retrieve relevant data. The literature search was performed up to March 2025 (without a lower time limit) and was restricted to publications in English.

The existence of additional relevant articles was determined by analyzing the reference lists of several of the articles retrieved. The following search terms were used: (Willebrand) AND ([epidural] OR [spinal] OR [neuraxial] OR [locoregional] OR [regional]) AND ([anesthesia] OR [anaesthesia]) AND ([delivery] OR [labor] OR [labour] OR [pregnancy] OR [obstetric] OR [childbirth] OR [pregnancy]). Inclusion criteria were as follows: NA (spinal or epidural) with VWF substitution at delivery in patients with VWD. Exclusion criteria included insufficient data (lack of information concerning treatments used, VWF doses, or VWF:Act levels or relating to acquired von Willebrand syndrome), or irrelevance to the research question.

## 3 Results

**Figure 1:**
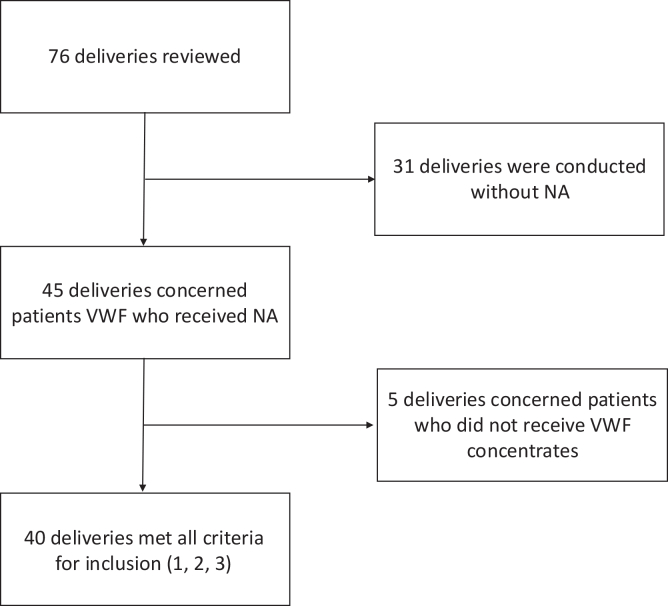
Flowchart of patient inclusion in the study. NA, neuraxial anesthesia; VWF, von Willebrand factor.

Of the 42 French hospital centers including a BDC, 8 provided data on a total of 76 deliveries for 44 patients with inherited VWD having been prescribed VWF concentrates in the maternity ward (Figure 1). Of the 76 deliveries reviewed, 36 were not included in the study for the following reasons: 31 deliveries were conducted without NA, and 5 deliveries concerned patients who did not receive any hemostatic treatment, either because VWF:Act was slightly > 0.5 IU/mL or owing to mismanagement.

Among the 40 deliveries corresponding to 32 patients meeting all inclusion criteria (1, 2, and 3), 36 took place in university hospital centers (UHCs) and 4 in non-UHCs (under the supervision of the referral UHC possessing a BDC). There was no multiple pregnancy. Median age at delivery was 32 years (IQR, 27.5-35.5).

Except for type 3 VWD, all types of VWD were represented in our study. Among the 32 patients included, the majority had type 1 (*n* = 12) or type 2A (*n* = 10) VWD, with the others manifesting type 2M (*n* = 8), type 2N (*n* = 1), or type 2B (*n* = 1) VWD.

Most patients had a history of surgery (26/32), with 20 (77%) having received a substitution treatment for their surgeries: 13 receiving VWF concentrate infusions, 4 desmopressin, 1 VWF concentrate infusion and tranexamic acid, and 2 desmopressin and tranexamic acid. Only 1 patient had experienced a bleeding complication, necessitating infusion of 1 unit of packed red blood cells, following a hysteroscopy; VWD had not been diagnosed in this patient at the time of the procedure.

Nine of the 40 deliveries (23%) were achieved by cesarean section. In 10 of 40 deliveries (25%), labor was induced; tears were described during 5 deliveries and episiotomy for 3 deliveries.

Before pregnancy, median laboratory values (range) were as follows: VWF:Act, 0.12 IU/mL (0.02-0.30); VWF:Ag, 0.21 IU/mL (0.06-0.96); FVIII, 0.4 IU/mL (0.08-0.82); fibrinogen, 3.07 g/L (2.05-5.31); and platelet counts, 270 G/L (199-401). Occlusion time values were abnormal in the 32 pregnancies for which values were available—epinephrine and ADP: > 300 and > 300 seconds in 15 patients; > 250 and > 250 seconds in 10 patients; > 200 and > 200 seconds in 4 patients; and < 200 and < 200 seconds in 3 patients, respectively.

**Figure 2:**
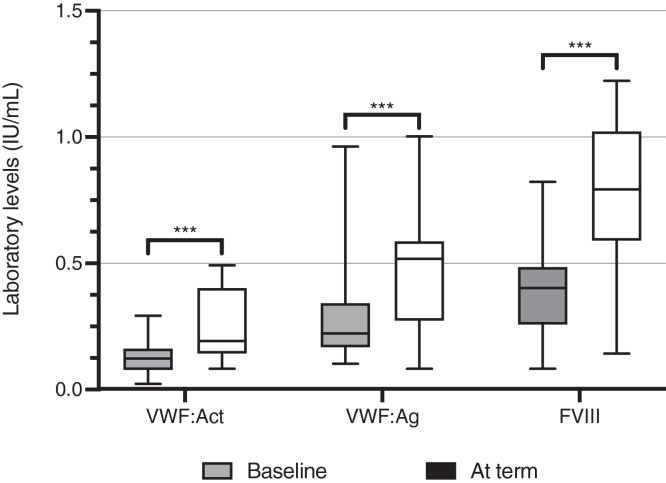
Median values of VWF:Ag, VWF:Act, and factor (F)VIII at baseline and at term for 40 pregnancies. The statistical significance of differences between medians was assessed by Wilcoxon signed-rank test. ∗∗∗*P* < .001. VWF, von Willebrand factor.

**Figure 3:**
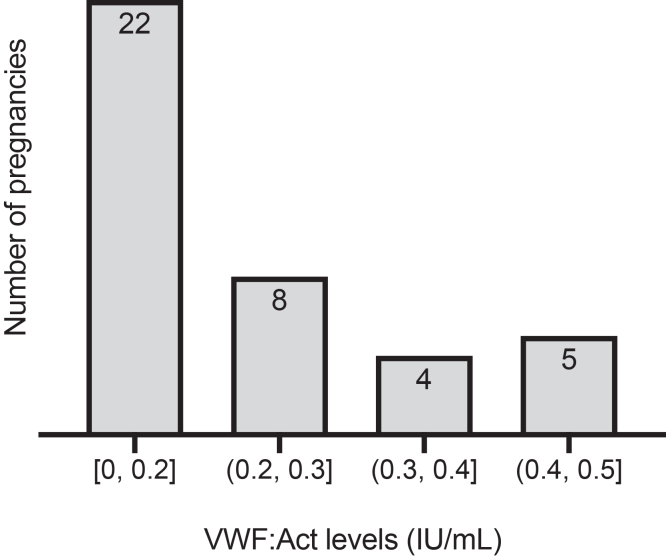
VWF:Act levels at term. Excluding the 1 patient with von Willebrand disease type 2N (VWF:Act = 76% and factor VIII = 26%). VWF, von Willebrand factor.

At the end of pregnancy, although median VWF:Act level increased significantly from 0.12 IU/mL (IQR, 0.02-0.30) to 0.19 IU/mL (IQR, 0.08-0.49; *P* < .001) and VWF:Ag increased from 0.21 IU/mL (IQR, 0.06-0.96) to 0.52 IU/mL (IQR, 0.08-1.00; *P* < .001) (Figure 2), VWF:Act levels remained < 0.2 IU/mL at term in 22 pregnancies (Figure 3). Median FVIII:C levels increased significantly from 0.4 IU/mL (IQR, 0.08-0.82) at baseline to 0.79 IU/mL (IQR, 0.11-1.80) at term (*P* < .001) (Figure 2). Median platelet count at term was 247 G/L (IQR, 133-407), and median fibrinogen level was 4.7 g/L (IQR, 3.1-6.2).

Both VWF and FVIII concentrates were used in 10 of 40 deliveries (25%). Among the 7 patients with FVIII:C levels of < 0.5 IU/mL at term, 4 received Wilfactin, 3 received Wilstart, and 2 received Voncento (Table 1). Only VWF concentrates were used for the other deliveries (30/40, 75%). In addition to VWF substitution, tranexamic acid was used for 17 deliveries (43%).

**Table 1:** Treatment and monitoring in patients with VWD with uncorrected VWF levels at term.

Pregnancy No.	VWF:Act level at term (IU/mL)	FVIII:C level at term (IU/mL)	Type of treatment	VWF dose used for first infusion (IU/kg)	Total VWF dose (IU/kg)	No. of infusions	VWF:Act level rtdafter first infusion (IU/mL)	FVIII level after first infusion (IU/mL)	Bleeding symptoms
1	0.1	0.22	Wilfactin	60	NA	NA	0.72	0.52	EBS
2	0.12	0.66	Veyvondi + Novoeight	46 and 38	NA	NA	0.75	1.39	EBS
3	0.47	NP	Wilstart	44	NA	NA	NP	0.96	EBS
4	0.4	0.96	Wilfactin	42	NA	NA	1.14	1.01	EBS
5	0.44	1.04	Veyvondi	38	NA	NA	1.47	1.54	EBS
6	0.1	1.12	Wilfactin	43	NA	NA	0.78	1.37	EBS
7	0.34	0.8	Wilfactin	40	NA	NA	1.47	0.78	EBS
8	0.17	1.22	Wilfactin	53	222	4	0.83	1.74	EBS
9	0.09	0.73	Wilfactin	48	290	6	0.44	1.5	EBS
10	0.31	0.79	Wilfactin	38	150	4	1.23	1.73	EBS
11	0.32	1.19	Wilfactin	36	180	5	1.16	1.51	EBS
12	0.37	0.27	Wilstart	36	36	1	0.82	1.23	EBS
13	0.24	1.02	Wilfactin	60	180	3	0.99	1.75	EBS
14	0.41	0.52	Veyvondi	55	167	3	1.16	1.73	EBS
15	0.15	0.24	Wilstart + Wilfactin	48	156	3	NP	NP	EBS
16	0.19	0.74	Veyvondi	47	141	3	0.79	1.08	Severe PPH (1800 mL), incomplete delivery
17	0.14	1.36	Wilfactin	60	176	3	1.17	NP	EBS
18	0.49	1.84	Veyvondi	41	145	5	1.5	NP	EBS
19	0.38	0.79	Veyvondi	40	NA	1	1.15	1.31	EBS
20	0.26	1.01	Wilfactin	60	180	3	0.89	2.16	EBS
21	0.39	0.82	Veyvondi	50	150	3	0.81	1.31	EBS
22	0.2	1.09	Wilfactin	50	717	18	0.52	1.14	Severe PPH (1800 mL), placenta previa
23	0.2	0.78	Veyvondi	34	84	3	0.76	1.27	Severe PPH (1200 mL), arterial wound
24	0.08	0.11	Voncento	55	451	12	0.51	0.84	Severe PPH (1000 mL), dystocia^a^
25	0.47	0.82	Wilfactin	30	NA	4	0.88	1.02	PPH (500 mL), Hysterorrhaphy
26	0.1	0.62	Wilfactin	50	620	16	NA	NA	No PPH but abnormal delayed bleeds^b^
27	0.18	0.84	Wilstart + Wilfactin	65	143	3	0.87	1.78	EBS
28	0.2	0.71	Wilstart + Wilfactin	40	289	12	0.57	1.13	EBS
29	0.15	0.49	Wilstart + Wilfactin	50	312	12	0.5	1.31	EBS
30	0.11	0.64	Wilfactin	50	258	11	2.29	1.58	EBS
31	0.16	0.91	Veyvondi	60	257	7	1.08	1.51	EBS
32	0.49	1.02	Wilfactin	12	11	1	NP	NP	EBS
33	0.4	0.97	Wilfactin	16	16	2	0.53	1.34	EBS
34	0.13	0.81	Wilfactin	50	354	9	NA	NA	EBS
35	0.11	0.21	Voncento	58	275	8	0.9	0.75	EBS
36	0.19	0.84	Voncento	64	109	8	0.66	1.17	EBS
37	0.28	0.52	Wilfactin	66	110	8	1.35	0.61	EBS
38	0.14	0.49	Wilfactin	54	189	6	1.32	0.71	EBS
39	0.45	1.01	Wilfactin	30	150	1	0.87	NA	EBS
40	0.25	0.78	Wilfactin	50	224	4	0.91	0.84	EBS

Data concerning the 40 deliveries of patients with VWD with uncorrected levels of VWF at term are presented. In pregnancy 1, patient received Wilfactin while having a low factor (F)VIII level, probably owing to erroneous prescription. This patient did not experience excessive bleeding symptoms. VWF:Act and FVIII levels were not always measured at peak levels but in the 24 hours following VWF concentrate infusion.

EBS, expected bleeding symptom; NA, not available; NP, not performed; PPH, postpartum hemorrhage; VWD, von Willebrand disease; VWF, von Willebrand factor.

a Patient 24 had a delayed term and manifested dystocia, leading to prolonged labor and finally cesarean section; this explains the large number of VWF concentrate infusions.

b Patient 26 underwent a prolonged hemostatic protocol owing to abnormal uterine bleeds necessitating rehospitalization. She received VWF concentrates during a total of 14 days from the day of delivery.

**Figure 4:**
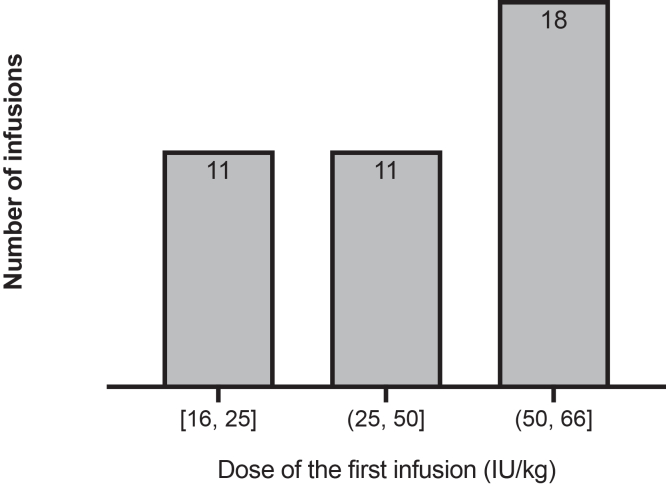
Initial von Willebrand factor dose infused (IU/kg).

The VWF doses in the first infusion varied (Figure 4), according to the VWF:Act level (Table 1). The median initial dose of VWF concentrates infused was 50 IU/kg (IQR, 16-66). Generally, the doses infused exceeded 30 IU/kg, enabling achievement of a VWF:Act level of > 0.5 IU/mL. The median number of VWF infusions was 4 (IQR, 1-18). If necessary, FVIII concentrates (median dose 20 IU/kg [IQR, 15-33]) were administered if FVIII:C levels were < 0.5 IU/mL (Table 1).

VWF:Act was measured at peak after the first VWF concentrate infusion for all patients with a VWF:Act value of 0.88 IU/mL (IQR, 0.44-2.29), all but 1 patient achieving a level of > 0.5 IU/mL. This patient received 5 subsequent doses to raise and maintain VWF:Act levels of > 0.5 IU/mL. In 7 patients, VWF:Act was measured 8 to 12 hours after VWF concentrate infusions until epidural catheter removal. The median FVIII level was 1.31 IU/mL (IQR, 1.01-1.51).

No SEH or ecchymosis related to NA during delivery was described in any of the 32 patients included (40 deliveries). Table 1 presents the data concerning patients with uncorrected levels of VWF at term reported by all centers. Pregnancies 9, 19, 26, and 34 were delivered at the same non-UHC, referring to a UHC. Concerning pregnancy 1, the patient received Wilfactin while presenting a low FVIII level, probably owing to erroneous prescription. This patient did not experience excessive bleeding symptoms.

Laboratory monitoring varied in timing according to the practice of each center. Assays were performed at peak or residual concentrations, depending on the availability of these assays in the laboratory and the delay involved in their implementation.

Five deliveries were not included in the study because the patients concerned did not meet criterion 2, ie, they did not receive VWF concentrates before NA (Figure 1). Among these 5 deliveries, 3 concerned patients who gave birth without the infusion of VWF concentrates, all of whom had a history of childbirth without such an infusion. Given the absence of bleeding in previous deliveries undergone by these patients, VWF concentrates were not infused prior to delivery. However, VWF concentrates were readily available in the maternity ward, and instructions were given for their administration in the event of bleeding. The other 2 patients not meeting criterion 2 did not receive VWF concentrates owing to mismanagement and miscommunication between the BDC physician and the anesthetist. Of these patients, 1 did not experience any adverse outcome, whereas the other sustained severe PPH requiring transfusion of 2 units of packed red blood cells and 1 unit of frozen fresh plasma. No SEH or ecchymosis was described for any of the 5 deliveries for which NA was performed without any VWF substitution.

Among the 32 patients included in the study, 9 (28%) had undergone at least 1 previous delivery (in total 10 deliveries) with NA prior to diagnosis of VWD. Their VWF levels on these occasions were consequently unknown. Considering that, in our study, the VWF:Act levels for these 9 patients ranged from 0.10 to 0.37 IU/mL, we can reasonably assume that their VWF levels were also low during these previous deliveries. However, NA use was uneventful in all 10 deliveries.

**Figure 5:**
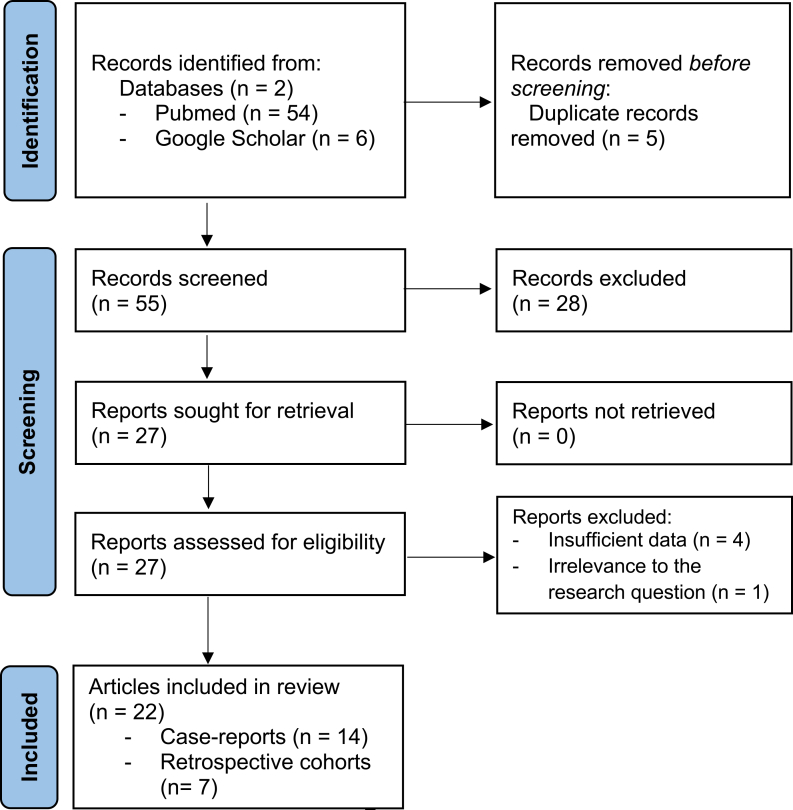
Flowchart of the literature review of publications concerning neuraxial anesthesia use during delivery in patients with von Willebrand disease. Established following PRISMA (Preferred Reporting Items for Systematic reviews and Meta-Analyses) guidelines.

As shown in the flowchart (Figure 5), our search of the 2 databases consulted yielded a total of 60 articles concerning the use of NA during delivery in patients with VWD (Table 2) [[10], [11], [12], [13], [14], [15], [16], [17], [18], [19], [20], [21], [22], [23], [24], [25], [26], [27], [28], [29], [30],^31^] . After deletion of 5 duplicate studies, 55 unique articles were retained for screening. This led to exclusion of 28 articles, primarily owing to insufficient data and irrelevance to the research question. Of the 27 full articles subsequently retrieved and assessed for eligibility, those authored by Nomura et al. [32], Skeith et al. [33], Malina et al. [34], Yousuf et al. [35], and Kazi et al. [36] were excluded, since they all lacked information about VWF levels, treatments used and dosage. All these articles nevertheless specified that no adverse effect related to NA was observed. In total, 22 articles, including a total of 199 pregnancies, met our inclusion criteria, describing the use of NA with VWF substitution at delivery in patients with VWD (Table 2, Figure 5). These articles comprised case reports (14/22) and retrospective cohorts (7/22), with 1 prospective study. The majority concerned patients with VWF levels of > 0.31 IU/mL at term. Only 8 articles of 22 included patients with uncorrected VWF levels at term. Among the 199 pregnancies described, 13 involved patients who benefited from NA while presenting VWF:Act levels of < 0.31 IU/mL before substitution. No SEH related to NA was described in any of the 199 pregnancies, including the 13 corresponding to patients with low VWF:Act levels but not receiving VWF substitution. Almost all articles lacked data on VWF monitoring and the timing of VWF assays.

**Table 2:** Review of literature.

Study	Design	No. of deliveries^a^	VWF type	Type of delivery	Type of neuraxial anesthesia	VWF:Act spontaneously corrected?	VWF:Act level at the end of pregnancy (IU/mL)	VWF:Act level before anesthesia (IU/mL)	Treatment during delivery	Dose at delivery	Remarks
Cohen et al. (1989) [10]	Case report	1	NA	Vaginal	Epidural	NA	NA	NA	Desmopressin	NA performed after epidural catheter placement	PT, PTT, platelet count, and fibrinogen normal
Sharpe et al. (2025) [11]	Retrospective cohort	8 (in 6 patients)	1 (5/6) and 2A (1/6)	Vaginal (3/8) and cesarean section (5/8)	Epidural (5/8) and spinal (3/8)	No in 5 patients, yes in 2 patients, NA for 1 patient	0.31 ± 0.17 (mean ± SD)	NA	VWF concentrates	NA	—
Kadir et al. (1998) [12]	Retrospective cohort	8	NA	Vaginal and cesarean section	NA (regional anesthesia)	Yes in 6 patients	>0.5	NA	None for 7 patients; VWF concentrates for 1 patient	NA	In 2 patients VWD was undiagnosed at time of delivery
Milaskiewicz et al. (1990) [13]	Case report	1	1	Cesarean section	Epidural	No	0.1	NA	None	—	—
Caliezi et al. (1998) [14]	Case report	1	3	Vaginal	NA (regional anesthesia)	No	<0.13	1.34	Intermediate purity FVIII concentrates	81 IU/kg	—
Jones et al. (1999) [15]	Case report	1	2A	Vaginal	Combined spinal and epidural	No	<0.25	1.12	Intermediate purity FVIII concentrates	30 IU/kg before NA then every 12 h for 48 h then once a day for 4 d	—
Cohen and Zada (2001) [16]	Case report	1	NA	Vaginal	Epidural	NA	NA	NA	Intermediate purity FVIII concentrates	Before epidural block and 12 h after block	PT, PTT, platelet count, and fibrinogen normal
Pérez-Barrero et al. (2003) [17]	Case report	1	1	Vaginal	Epidural	Yes	NA	NA	Desmopressin	0.3 μg/kg before NA	PT, PTT, platelet count, and fibrinogen normal
Suddeth et al (2003) [18]	Retrospective cohort	34	NA	NA	Epidural	NA	NA	NA	Desmopressin for 5 deliveries	NA	—
Varughese and Cohen (2007) [19]	Retrospective cohort	17 (15 patients)	1 (14/15) and 2A (1/15)	Vaginal (13/17) and cesarean section (4/17)	Epidural	Yes	NA	2 and 2.6 (available for only 2 deliveries)	None	/	VWF:Act > 0.5 IU/mL at base level in 10 patients^b^
Butwick and Carvalho (2007) [20]	Case report	1	1	Vaginal	Combined spinal epidural	Yes	>2.4	2.5	Desmopressin	0.3 μg/kg before NA	—
Marrache et al. (2007) [21]	Retrospective cohort	9 (6 patients)	1	Vaginal (7/9) and cesarean section (2/9)	Epidural	Yes	1.4 ± 0.79	NA	None	—	—
Kailash and Wilkerson (2009) [22]	Case report	1	1	Cesarean section	Epidural	Yes	0.99	NA	None	—	—
Cata et al. (2009) [23]	Case report	1	2M	Cesarean section	Spinal	NA	NA	NA	None	—	No VWF tests were performed before delivery; after delivery VWF:Act = 0.83 IU/mL
Schuitemaker, Requena et al. (2010) [24]	Case report	1	1	Vaginal	Combined spinal epidural	Yes	0.88	NA	None	—	—
Sood et al. (2010) [25]	Prospective study	8	1	Vaginal and cesarean section	Epidural (7/8) and spinal anesthesia (1/8)	Yes	1.3 ± 0.48	NA	None	—	—
Amorde et al. (2011) [26]	Case report	1	2A	Cesarean section	Epidural	No	<0.1	NA	VWF + FVIII concentrates	50 IU/kg 1 h before epidural placement, 25 IU/kg before cesarean section, and 25 IU/kg every 12 h until postpartum day 4	—
Lagarrigue et al. (2013) [27]	Case report	1	2B	Vaginal	Epidural	Yes	2.9	3	VWF concentrates	50 IU/kg before NA and before catheter removal, 40 IU/kg H+12 postpartum and 20 at H24	—
Parker et al. (2018) [28]	Case report	2 (1 patient)	3	Cesarean section	Spinal	No	Absent and <0.05	1 and 0.67	VWF + FVIII concentrates	3 IU/kg/h continuous infusion 3 d before delivery and then 2 IU/kg/h; 3 IU/kg/h for the second delivery	Tranexamic acid was also used
Prior et al. (2020) [29]	Case report	1	1C	Vaginal	Epidural	No	0.23	NA	VWF + FVIII concentrates	2 doses of 75 IU/kg: 1 before NA and 1 during labor	—
Reale et al. (2021) [30]	Retrospective cohort	94	1 (79/94), 2 (3/94), and NA (12/94)	Vaginal (53/94) and cesarean section (41/94)	Epidural (55/94), spinal (27/94), and combined (12/97)	NA	NA	NA	Desmopressin (for 21 patients) and VWF + FVIII concentrates (for 6 patients)	Before NA: 0.3 μg/kg (desmopressin) and 50 IU/kg (VWF+FVIII concentrates)	—
Yi et al. (2025) [31]	Retrospective cohort	6	1 (4/6), 2 (1/6) and 2N (1/6)	Vaginal (1/6) and unknown (5/6)	Epidural (4/6) and unspecified (2/6)	No	0.49 ± 0.26	NA	None (5/6) and VWF concentrates (1/6)	NA	—

NA, not available; PT, prothrombin; PTT, partial thromboplastin time; VWD, von Willebrand disease; VWF, von Willebrand factor.

a Number of patients with VWD deliveries during which neuraxial anesthesia was performed.

b Available for only 2 patients.

## 4 Discussion

The use of NA in patients with VWD and uncorrected VWF levels at the end of pregnancy has been a matter of discussion for several years now, with the first article concerning this issue being published in 1989 [10]. Our literature review underlines the absence of practice standardization across hospital centers. Up to early 2025, the question of NA use for delivery in patients with VWD with nonspontaneously corrected VWF level was specifically raised in only 7 articles, all case reports except for 1 prospective study [11], which also included women with corrected VWF levels. NA was found to be safe in all the cases described.

The recommendations concerning NA use in patients with VWD vary according to the medical academic society concerned. The US National Heart, Lung, and Blood Institute guidelines published in 2008 emphasize that there is no consensus regarding the VWF:Act and FVIII:C levels required to ensure the safety of NA use for delivery. Nevertheless, they indicate that NA may be safely implemented when these levels exceed 0.5 IU/mL [37]. The recommendation for clinical management states, “if VWF:RCo and FVIII levels can be monitored and maintained > 50 IU/dL during labor and delivery, and no other coagulation defects are present, then regional anesthesia may be considered.” This recommendation is nevertheless rated as grade C and is mainly supported by the study of Kadir et al. [12], which described 8 patients (of a cohort of 31 with VWD deficiency) benefiting from NA whose VWF levels had been corrected spontaneously by the end of pregnancy.

The UK national guideline issued in 2014 does not recommend NA in women with VWF:Act levels < 0.5 IU/mL at the end of pregnancy, “irrespective of whether VWF activity has been restored to apparently normal levels” [38], stating that restoration of normal VWF levels with VWF concentrates cannot be considered reliable. In 2017, the United Kingdom Haemophilia Centre Doctors’ Organisation (UKHCDO) and the Royal College of Obstetricians and Gynaecologists (RCOG) issued a combined guideline on the management of inherited bleeding disorders in pregnancy [39]. While this guideline considers NA to be safe in women with type 1 VWD when VWF:Act is > 0.5 IU/mL, it counsels its avoidance in patients with type 2 or 3 VWD. According to the authors, hemostasis “may not normalize even with replacement therapy because of associated platelet dysfunction.” The evidence level of this recommendation is rated as IV (ie, low).

The French national diagnosis and care protocol (PNDS) published in 2021 states that NA is possible in patients with type 1 VWD if FVIII and VWF levels are > 0.5 IU/mL at 34 weeks of amenorrhea [40], implying that NA is not possible in the absence of spontaneous correction. This protocol specifically does not recommend NA for patients with type 2 VWD and contraindicates its use in patients with type 3 VWD. The guidelines of the French Society of Anesthetics (SFAR) are even more restrictive since they contraindicate NA use in the context of any hemostatic disorder.

Currently, the International Society on Thrombosis and Haemostasis (ISTH) international guideline suggests, “targeting a VWF activity level of 0.50 to 1.50 IU/mL over targeting an activity level of > 1.50 IU/mL to allow neuraxial anesthesia” [9]. However, this recommendation is based on 5 studies with a low certainty of evidence, with NA in all these studies being uneventful. Three of these studies concerned solely hemophilic patients [[41], [42], [43]], and the other 2 studies (Kadir et al. [12] and Chi et al. [44]) included either patients with VWD or women with other inherited bleeding disorders. These studies described 14 and 8 cases of NA use, respectively, in patients with inherited VWD. In all these patients, NA was performed only if VWF levels were > 0.5 IU/mL.

Overall, the limitation of the ISTH guideline is that it is based on only 2 studies including 22 patients in total. Furthermore, in both these studies, only patients with spontaneously corrected VWF levels at the end of pregnancy received NA.

Moreover, in the 2021 ISTH recommendation [9], it is not specified whether VWF levels must be spontaneously corrected or can be corrected by pharmaceutical substitution. The ISTH Scientific and Standardization Committee (SSC) on VWF conducted a Delphi consensus study in 2025 on NA in adults with platelet disorders and coagulation defects. The same VWF:Act threshold of 0.5 IU/mL, achieved after substitution if necessary, was highlighted as the minimum level of VWF:Act required for NA [45].

In our study, 31 patients presented a VWF:Act level of > 0.5 IU/mL after VWF substitution, with only 1 patient manifesting a lower level (0.44 IU/mL). NA was uneventful, and the safety of this threshold corroborates the international guidelines.

However, the variety of practices observed in our study reflects the variability of guidelines on VWF substitution for NA. Our study demonstrates that practices vary across France, only 8 of the 46 hospitals including a BDC performing NA after VWF concentrate infusion in VWD women whose VWF levels had not been spontaneously corrected by the end of pregnancy.

Practices also vary at the international level. In 2022, Lavin et al. [5] surveyed worldwide practices for the management of pregnant women with VWD. In reply to the question “Can neuraxial anesthesia be offered to women with VWD at delivery?”, the 108 physicians surveyed gave various answers according to VWD type. Approximately one-third considered NA unsuitable for patients with type 2 or 3 VWD, irrespective of VWF levels. Barely half of the responders were in favor of NA if the levels of VWF:Act (or VWF:Act and VWF:Ag) were > 0.5 IU/mL.

Our study also highlighted the variability of treatment decisions according to the hospital center, the hemostasis specialist, and the previous deliveries of each patient. To the best of our knowledge, this is the largest study (32 patients; 40 deliveries) focusing on NA performed after pharmaceutical correction of VWF in patients in whom spontaneous correction of VWF levels had not occurred by the end of pregnancy.

The results of our study show that correcting VWF:Act ± VIII deficiencies by infusion of VWF ± FVIII concentrates could ensure the safety of NA, provided that this treatment is closely monitored. Interestingly, in our study, 22 patients still showed very low VWF:Act levels at term (< 0.2 IU/mL). Overall, VWF substitution was effective, as the attainment of a median level of VWF:Act of 0.88 IU/mL (IQR, 0.44-2.29; with only 1 value <0.5) was sufficient to preclude the hemorrhagic risk related to NA. Although healthy pregnant women generally have VWF:Act levels exceeding 0.5 IU/mL at delivery, the levels observed were sufficient to avoid SEH.

Although the deliveries in non-UHCs (concerning pregnancies 9, 19, 26, and 34) were uneventful in our study, it is preferable for patients with VWD to deliver in a hospital with a BDC, an intensive care unit, and easy access to hemostasis tests and VWF concentrates. Some patients had a history of delivery with NA before VWD diagnosis, and this situation may not be uncommon. Broadly speaking, some patients with undiagnosed VWD deficiency have normal FVIII levels and, therefore, display normal activated partial thromboplastin time results in screening tests. It is likely that VWF levels in some of those patients have not been fully corrected by the end of pregnancy. Consequently, anesthetists may use NA for these patients while unaware of a hemostasis abnormality not detected by routine coagulation tests, hence the importance of medical examination, exploration of personal and familial history of signs of bleeding, and determination of hemorrhagic scores. In patients having previously manifested signs of bleeding, even when activated partial thromboplastin time and FVIII levels are normal, the possible presence of VWD could be checked by performing a platelet function assay and determining VWF:Act and VWF:Ag levels, as well as the VWF:Act/VWF:Ag ratio.

Measurement of VWF levels at the end of pregnancy and at delivery can ensure that the target of VWF:Act > 0.5 IU/mL has been reached. Analysis of the recuperation rate after infusion of VWF concentrates performed a few weeks before the time of delivery could allow precise selection of the first dose of VWF concentrates.

One of the benefits of performing NA in these patients is the possibility of avoiding general anesthesia when a cesarean section is needed. General anesthesia carries a higher risk of maternal complications (respiratory issues and airway management) and aspiration pneumonitis [46]. General anesthesia is widely avoided for delivery but is the only alternative for cesarean section when NA is contraindicated [47,48]. Patients with VWD with low VWF levels at term could benefit from spinal anesthesia without any additional hemorrhagic risk and thereby avoid the possible need for general anesthesia during delivery.

To ensure the safe use of NA for patients with VWF deficiency whose VWF levels have not been spontaneously corrected by the end of pregnancy, we suggest the following:

- Performing laboratory tests (VWF:Act, VWF:Ag, FVIII:C, fibrinogen level, and platelet count) at the end of pregnancy to evaluate the bleeding risk and the need for substitutive treatment.
- Following a personalized hemostasis protocol prepared by the hemostasis specialist in collaboration with the anesthetist, the gynecologist, and the hospital pharmacist.
- Performing a brief evaluation of the pharmacokinetics of the VWF concentrate 3 to 4 weeks before the time of delivery, with the objective of establishing VWF levels at peak, determining the recuperation rate, and facilitating patient management on the day of delivery.
- Planning delivery in a hospital center with easy access to VWF:Act and FVIII tests and to VWF concentrates. VWF concentrates could be prepositioned in the delivery unit on arrival of the patient.
- Entrusting NA to a trained anesthesiologist.
- Timing VWF infusions 30 minutes to 1 hour before NA.
- Maintaining VWF:Act level > 0.5 IU/mL throughout delivery. In the case of epidural anesthesia, this level should be sustained for at least 6 hours after catheter removal, in accordance with international recommendations [7]. Monitoring should be implemented, if possible, just before and 1 hour after the first VWF concentrate infusion (especially if no pharmacokinetic evaluation has been performed previously). VWF:Act level should be monitored every 8 to 12 hours.
- Timing subsequent VWF infusions in accordance with VWF:Act laboratory test results.
- Using VWF:Act level as a reference rather than VWF:Ag, as the latter level tends to be higher, especially in type 2 VWD (Figure 2). Lavin et al. [5] showed that ∼15% to 20% of physicians based their decisions on VWF:Ag level only, rather than on VWF:Act level as generally recommended.
- Using FVIII level as a reference in VWD type 2N.
- Systematically taking into consideration other possible causes of bleeding with regard to patients with corrected levels of FVIII and VWF who, nevertheless, experience a hemorrhagic complication [13,49,50].

**Figure 6:**
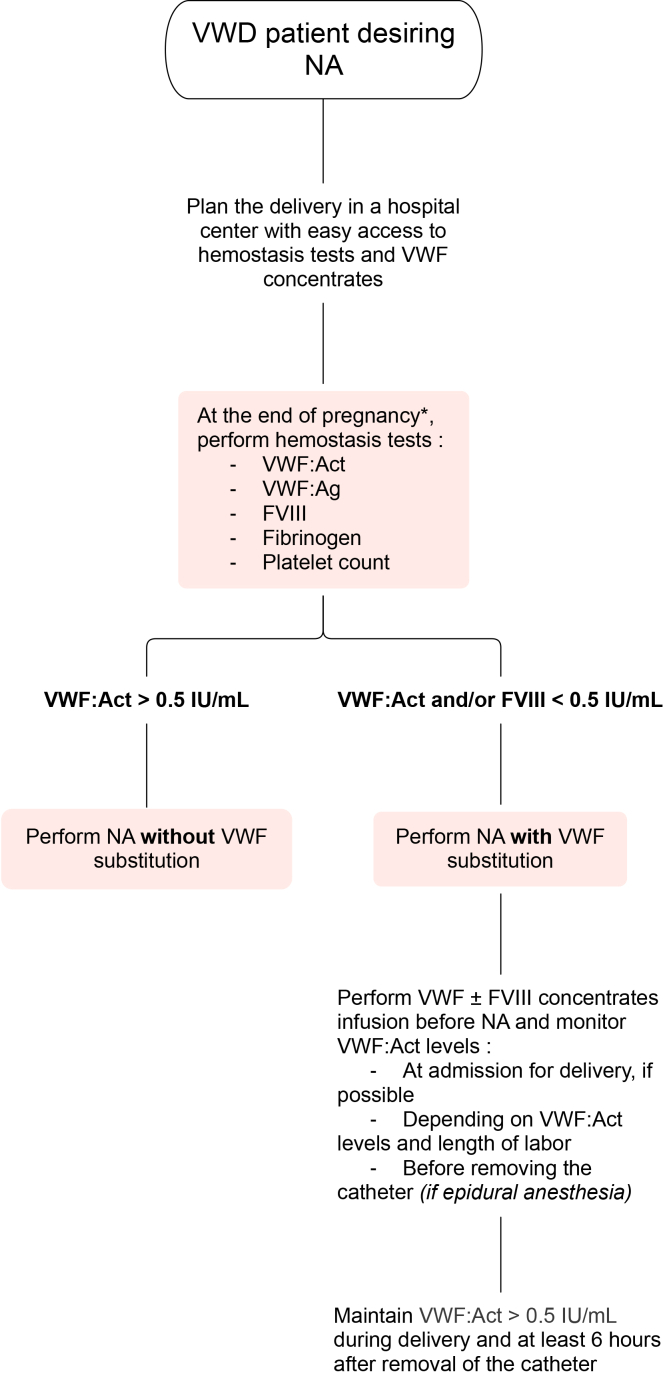
Flowchart for decision on whether or not to perform NA. ∗End of pregnancy, that is, third trimester. F, factor; NA, neuraxial anesthesia; VWD, von Willebrand disease; VWF, von Willebrand factor.

We propose a flowchart (Figure 6) to guide the decision-making process regarding anesthesia. The main limitation of our study is its retrospective nature, resulting in some missing data.

In conclusion, it is reasonable to assume that in patients whose VWF:Act levels remain uncorrected (< 0.5 IU/mL) at the end of pregnancy, well-conducted monitoring of VWF doses and adequate VWF substitution to maintain VWF:Act level > 0.5 IU/dL could ensure the safety of NA without any increase in hemorrhagic risk. Interdisciplinary collaboration between obstetricians, hematologists, and anesthesiologists is crucial for developing tailored approaches that prioritize maternal and newborn safety while assuring effective pain management during delivery.

## References

[1] Sadler J.E., Mannucci P.M., Berntorp E., Bochkov N., Boulyjenkov V., Ginsburg D. (2000). Impact, diagnosis and treatment of von Willebrand disease. Thromb Haemost.

[2] Sadler J.E. (1994). A revised classification of von Willebrand disease: for the Subcommittee on von Willebrand Factor of the Scientific and Standardization Committee of the International Society on Thrombosis and Haemostasis. Thromb Haemost.

[3] Hellgren M. (2003). Hemostasis during normal pregnancy and puerperium. Semin Thromb Hemost.

[4] Castaman G., James P.D. (2019). Pregnancy and delivery in women with von Willebrand disease. Eur J Haematol.

[5] Lavin M., Sánchez Luceros A., Kouides P., Abdul-Kadir R., O’Donnell J.S., Baker R.I. (2022). Examining international practices in the management of pregnant women with von Willebrand disease. J Thromb Haemost.

[6] Al-Mutair A., Bednar D.A. (2010). Spinal epidural hematoma. Am Acad Orthop Surg.

[7] Practice Bulletin No. 177: Obstetric Analgesia and Anesthesia (2017). Obstet Gynecol.

[8] Liu H., Brown M., Sun L., Patel S.P., Li J., Cornett E.M. (2019). Complications and liability related to regional and neuraxial anesthesia. Best Pract Res Clin Anaesthesiol.

[9] Connell N.T., Flood V.H., Brignardello-Petersen R., Abdul-Kadir R., Arapshian A., Couper S. (2021). ASH ISTH NHF WFH 2021 guidelines on the management of von Willebrand disease. Blood Adv.

[10] Cohen S., Daitch J.S., Amar D., Goldiner P.L. (1989). Epidural analgesia for labor and delivery in a patient with von Willebrand’s disease. Reg Anesth.

[11] Sharpe E.E., Pompeian R.J., Marshall A.L. (2025). Recombinant von Willebrand factor use in obstetric anesthesia. Proc (Bayl Univ Med Center).

[12] Kadir R.A., Lee C.A., Sabin C.A., Pollard D., Economides D.L. (1998). Pregnancy in women with von Willebrand’s disease or factor XI deficiency. Br J Obstet Gynaecol.

[13] Milaskiewicz R.M., Holdcroft A., Letsky E. (1990). Epidural anaesthesia and Von Willebrand’s disease. Anaesthesia.

[14] Caliezi C., Tsakiris D.A., Behringer H., Kühne T., Marbet G.A. (1998). Two consecutive pregnancies and deliveries in a patient with von Willebrand’s disease type 3. Haemophilia.

[15] Jones B.P., Bell E.A., Maroof M. (1999). Epidural labor analgesia in parturient with von Willebrand’s disease type IIA and severe preeclampsia. Anesthesiology.

[16] Cohen S., Zada Y. (2001). Neuroaxial block for von Willebrand’s disease. Anaesthesia.

[17] Pérez-Barrero P., Gil L., Martínez C., Bueno A.B., Casado A.I., Oro J. (2003). [Treatment with desmopressin before epidural anesthesia in a patient with type I von Willebrand disease]. Rev Esp Anestesiol Reanim.

[18] Suddeth B.H., Schmalenberger K.P., Mandell G.I., Golebiewsky K.A. (2003). Von Willebrand’s disease and regional anesthesia in the parturient. Anesthesiology.

[19] Varughese J., Cohen A.J. (2007). Experience with epidural anaesthesia in pregnant women with von Willebrand disease. Haemophilia.

[20] Butwick A.J., Carvalho B. (2007). Neuraxial anesthesia for cesarean delivery in a parturient with type 1 von Willebrand disease and scoliosis. J Clin Anesth.

[21] Marrache D., Mercier F.J., Boyer-Neumann C., Roger-Christoph S., Benhamou D. (2007). Epidural analgesia for parturients with type 1 von Willebrand disease. Int J Obstet Anesth.

[22] Kailash F., Wilkerson D. (2009). von Willebrand disease, pregnancy and neuraxial anesthesia: a multi-disciplinary approach for successful regional anesthesia. J Ark Med Soc.

[23] Cata J.P., Hanna A., Tetzlaff J.E., Bishai A., Barsoum S. (2009). Spinal anesthesia for a cesarean delivery in a woman with type-2M von Willebrand disease: case report and mini-review. Int J Obstet Anesth.

[24] Schuitemaker Requena J.B., Lopez Pantaleon L.A., Rodriguez Perez C.L., Tejada Perez R., De Armas M.N., Emperador F. (2010). Labor analgesia in patient with von Willebrand type I disease. Reg Anesth Pain Med.

[25] Sood S.L., James A.H., Bolgiano D., Ragni M.V., Shapiro A.D., Shihong I. (2010). Pregnancy in type 1 von Willebrand disease: a prospective study of VWF levels and risk factors for bleeding. Blood.

[26] Amorde R.W., Patel S.N., Pagel P.S. (2011). Management of labor and delivery of a patient with von Willebrand disease type 2A. Int Anesthesiol Clin.

[27] Lagarrigue J., Richez B., Julliac B., Saltel L., Nurden P., Sztark F. (2013). Analgésie péridurale obstétricale et maladie de Willebrand de type 2B. Ann Fr Anesth Reanim.

[28] Parker J.W., James P.D., Haley S.L. (2019). Spinal anesthesia in 2 consecutive cesarean deliveries in a parturient with type 3 von Willebrand disease: a case report. A A Pract.

[29] Prior C., Sims K., Seligman K., Jackson S., Chau A. (2020). Peripartum management of a parturient with type 1C (clearance) von Willebrand disease. Int J Obstet Anesth.

[30] Reale S.C., Farber M.K., Lumbreras-Marquez M.I., Connors J.M., Carabuena J.M. (2021). Anesthetic management of von Willebrand disease in pregnancy: a retrospective analysis of a large case series. Anesth Analg.

[31] Yi Y., Niu B., Duffett L., El-Chaâr D., Tinmouth A., Wang T.-F. (2025). Neuraxial analgesia in pregnant individuals with bleeding disorders: a retrospective descriptive study of obstetric anesthesia practices and outcomes. Res Pract Thromb Haemost.

[32] Nomura R.M.Y., Igai A.M.K., Zugaib M. (2008). Complicações do parto e resultados perinatais em gestantes portadoras da doença de von Willebrand. Rev Assoc Med Bras (1992).

[33] Skeith L., Goodyear M.D., Rydz N., Poon M.-C. (2014). Epidural analgesia use in women with von Willebrand disease. Blood.

[34] Malina M., Abdul-Kadir R., Pollard D., Siebert M., Halimeh S. (2015). Severe von Willebrand disease (VWD) in pregnancy and childbirth: a case series. Thromb Res.

[35] Yousuf S., Cohen A.J., Eris E., Astsaturov A. (2017). A single institutional study on pregnancy outcomes in patients with von Willebrand disease. Blood.

[36] Kazi S., Malinowski A.K., Whitehead C., Kuo K.H.M., Harrington L., Watts N. (2018). Successful management of pregnancy with severe von Willebrand disease in a Jehovah’s witness with recombinant von Willebrand factor. Blood.

[37] Nichols W.L., Hultin M.B., James A.H., Manco-Johnson M.J., Montgomery R.R., Ortel T.L. (2008). von Willebrand disease (VWD): evidence-based diagnosis and management guidelines, the National Heart, Lung, and Blood Institute (NHLBI) Expert Panel report (USA). Haemophilia.

[38] Laffan M.A., Lester W., O’Donnell J.S., Will A., Tait R.C., Goodeve A. (2014). The diagnosis and management of von Willebrand disease: a United Kingdom Haemophilia Centre Doctors Organization guideline approved by the British Committee for Standards in Haematology. Br J Haematol.

[39] Management of inherited bleeding disorders in pregnancy (2017). Green-Top Guideline No. 71 (joint with UKHCDO). Br J Obstet Gynaecol.

[40] Centre de Référence de la Maladie de Willebrand (2021). https://www.has-sante.fr/upload/docs/application/pdf/2021-02/maladie_de_willebrand_-_pnds.pdf.

[41] Kadir R.A., Economides D.L., Braithwaite J., Goldman E., Lee C.A. (1997). The obstetric experience of carriers of haemophilia. Br J Obstet Gynaecol.

[42] Chi C., Lee C.A., Shiltagh N., Khan A., Pollard D., Kadir R.A. (2008). Pregnancy in carriers of haemophilia. Haemophilia.

[43] Duggan S., Dockrell L., McCaul C. (2017). A retrospective, single-centre study of central neuraxial blockade in haemophilia carrier parturients. Ir J Med Sci.

[44] Chi C., Lee C.A., England A., Hingorani J., Paintsil J., Kadir R.A. (2009). Obstetric analgesia and anaesthesia in women with inherited bleeding disorders. Thromb Haemost.

[45] Peterson W., Martin R., Arnold D., Carvalho B., Cuker A., Gadsden J. (2025). Delphi consensus recommendations for neuraxial anesthesia in adults with platelet disorders and coagulation defects: communication from the ISTH SSC Subcommittee on von Willebrand Factor. J Thromb Haemost.

[46] Ring L., Landau R., Delgado C. (2021). The current role of general anesthesia for cesarean delivery. Curr Anesthesiol Rep.

[47] Delgado C., Ring L., Mushambi M.C. (2020). General anaesthesia in obstetrics. BJA Educ.

[48] Chaggar R., Campbell J. (2017). The future of general anaesthesia in obstetrics. BJA Educ.

[49] Punt M., van Leusden F., Bloemenkamp K., Coppens M., Driessens M., Heubel-Moenen F. (2024). Primary postpartum hemorrhage in women with von Willebrand disease and carriers of hemophilia: a retrospective analysis. Res Pract Thromb Haemost.

[50] Kazi S., Arusi I., McLeod A., Malinowski A.K., Shehata N. (2022). Postpartum hemorrhage in women with von Willebrand disease: consider other etiologies. J Obstet Gynaecol Can.

